# Navigating Professional Code of Conduct Among Undergraduate Dental Students in Pakistan: A Qualitative Study

**DOI:** 10.1002/hsr2.73408

**Published:** 2026-10-23

**Authors:** Sarah Ali, Syed Ali Ayub, Shahzad Ahmad, Riaz Shahbaz Janjua, Jamilah Janjua, Shazia Iqbal

**Affiliations:** ^1^ HBS Medical and Dental College Islamabad Pakistan; ^2^ The University of Buckingham Buckingham UK

**Keywords:** dental professionalism, dental students, hidden curriculum, moral distress, Pakistan, professional ethics, qualitative research, template Analysis

## Abstract

**Background:**

Professionalism in dentistry extends beyond technical competence to include accountability, respect for patients and colleagues, non‐maleficence, informed consent, communication, fairness, and conduct consistent with professional standards. In Pakistan, these expectations are shaped by international bioethical principles, national professional regulation, and local social and religious contexts.

**Aim:**

To explore undergraduate dental students' experiences of, or observations of, events they perceived as unethical or inconsistent with professional conduct during clinical/workplace learning, and to understand the forms and meanings of these experiences.

**Methods:**

A qualitative narrative inquiry was conducted over 6 months (November 2020–April 2021) at two dental colleges in Peshawar, Pakistan, one public and one private. Purposive sampling was used to recruit clinically exposed undergraduate dental students. Fifty students were approached and 37 participated in semi‐structured interviews. Data were analyzed using Template Analysis. The report follows the COREQ framework and makes the analytic, reflexive, and trustworthiness procedures explicit.

**Results:**

Participants had a mean age of 20.9 ± 1.96 years (range 17–25); 29 (78.4%) were female and 8 (21.6%) male. Four interconnected themes were identified: unprofessionalism, harassment, poor communication, and discrimination. The accounts included concerns about infection control, empathy, accountability for clinical error, dishonesty, physical/verbal/emotional harassment, inadequate instruction, failures of informed consent, abuse of power, victimization, and targeting.

**Conclusion:**

Students' accounts point primarily to breaches of professional conduct and to tensions between formal ethical expectations and the hidden curriculum of clinical learning. Professionalism education should therefore be embedded longitudinally in dental curricula and reinforced through faculty role‐modeling, safe reporting pathways, supervised clinical practice, and context‐sensitive ethical reasoning that can hold universal duties together with legitimate cultural and religious considerations.

## 1 Introduction

Professional morality in healthcare is not limited to compliance with a written code. Beauchamp and Childress distinguish a broadly shared common morality from particular moralities associated with roles, communities and practices, while also emphasizing that moral justification requires specification and balancing when general norms meet particular circumstances [1]. Their account is useful for professionalism because a professional code translates broad moral commitments such as respect for persons, non‐maleficence, beneficence and justice into role‐specific duties, but it cannot eliminate the need for judgment in context.

Common morality refers to broadly shared ethical obligations such as respect for persons, avoidance of harm, promotion of welfare, and fairness. In healthcare, these commitments are often expressed through the four principles of autonomy, beneficence, non‐maleficence, and justice. In contrast, particular moralities arise within specific professions, cultures, religions, or communities and shape how ethical responsibilities are interpreted in practice. Beauchamp and Childress argue that ethical decision‐making therefore requires specification and balancing, particularly when principles appear to conflict in real‐world clinical situations. This framework is relevant to Pakistani dental education, where professional codes interact with local social, cultural, and religious values.

This distinction is particularly relevant to Pakistan. The Pakistan Medical and Dental Council (PM & DC) is the national regulator for medical and dental practitioners, and the PM & DC Act requires registered practitioners to comply with the Council's code of medical ethics [2]. The regulatory framework therefore gives professional ethics a legal and disciplinary dimension rather than treating professionalism as a purely educational ideal. The Council's revised ethics framework, enforced in 2026, further places patient safety, dignity, confidentiality, informed consent, accountability, non‐discrimination and equitable care at the center of professional conduct [3]. These principles are directly relevant to the experiences described by dental students in this study.

At the same time, professional morality in Pakistan operates within a plural social environment in which family relationships, authority structures, religion and social expectations may influence ethical reasoning [4]. Research from Pakistan has documented tensions between individual autonomy and family involvement in decision‐making and has emphasized the importance of contextualizing informed consent and other ethical practices rather than assuming that a single culturally neutral model will automatically translate into practice. Islamic bioethical scholarship also demonstrates substantial overlap between principles such as protection of life, avoidance of harm, justice, dignity and beneficence and contemporary biomedical ethics, while recognizing that their moral grounding and application may differ [5].

This does not justify a simple binary between “Western” and “Islamic” ethics. Such a binary risks treating both traditions as internally homogeneous. A more useful approach is specification: universal moral commitments can be interpreted and operationalized through the social, legal and religious circumstances in which professional practice occurs. For example, truthfulness and disclosure remain important professional duties, but the manner, timing and relational setting of disclosure may require careful balancing where family involvement and social harmony are important. The ethical question is therefore not whether one morality should replace another, but how competing or overlapping norms can be specified without compromising core protections against harm, deception, coercion and discrimination.

The dental profession provides a particularly appropriate setting for this inquiry. Dental students encounter patients during invasive procedures, obtain or observe informed consent, participate in infection‐control practices, learn through close faculty–student relationships, and work within a pronounced hierarchy [6]. The FDI International Principles of Ethics for the Dental Profession are explicitly dental‐specific and state that international principles cannot cover every local tradition, legislation or circumstance [7]. In Pakistan, the PM & DC framework applies directly to registered medical and dental practitioners, while the dental curriculum prepares students for the professional responsibilities that follow registration [3]. Thus, the dental code is not selected arbitrarily: it is the profession‐specific normative framework most directly connected to the clinical situations described by dental students.

The study addresses a gap between formal professionalism teaching and the hidden curriculum of clinical practice. Previous work has identified ethical dilemmas among dental students and highlighted the need for professionalism education, but less is known about how Pakistani dental undergraduates experience conduct that they perceive as inconsistent with professional obligations [8]. A qualitative approach was selected because the research question concerns experiences, meanings, perceptions and contextualized accounts rather than the estimation of prevalence or testing of predetermined causal associations.

## 2 Aim and Objectives

To explore undergraduate dental students' experiences of, or observations of, situations they perceived as unethical or inconsistent with professional conduct during workplace/clinical learning in Pakistan, and to examine how these experiences were understood in relation to professional responsibilities.

## 3 Methodology

A qualitative narrative inquiry design was used, appropriate for exploring experiences through concrete events and interactions in the clinical learning environment. This design enabled participants to describe what happened, who was involved, how they interpreted events, and how these shaped their understanding of professional conduct. A qualitative approach was chosen over a survey to gain explanatory depth and contextual meaning. Analysis employed Template Analysis, a flexible thematic method allowing both a priori and inductive themes.

Reporting was guided by the 32‐item COREQ checklist for qualitative interviews, used as a transparency framework rather than a claim of validity [9]. The completed checklist is provided in Appendix A (Table A1).

The study ran from November 2020 to April 2021 at two dental colleges in Peshawar (one public, one private), selected to capture variation in institutional context. The setting involved undergraduate clinical training with interactions among faculty, trainees, patients, and healthcare staff. Participants were second‐ to fourth‐year dental students with clinical exposure; 50 were approached, 37 completed semi‐structured interviews, and reasons for non‐participation were not systematically documented.

Purposive sampling ensured relevant clinical experience. Recruitment invitations were disseminated via email, WhatsApp, Facebook, and notice boards; social media was used only for recruitment, not data collection. Participation was voluntary, with written informed consent obtained. To mitigate risks of perceived pressure or privacy concerns, invitations emphasized voluntariness, confidentiality, and research use of responses. Identifiers were removed or generalized in reporting.

Data generation used a semi‐structured guide of five core questions with prompts, refined after expert review. Interviews were conducted with 37 students. The record does not specify interview duration, setting, or language for each case. Given the sensitive topics (harassment, abuse of power, misconduct), participants were reminded of their right to decline or withdraw. Quotations were anonymized.

Data were analyzed using Template Analysis [10, 11]. An initial template was developed from study objectives, interview guide, ethics literature, and PM & DC/FDI frameworks, comprising four provisional domains: unprofessional conduct/patient safety; harassment/hierarchy; communication/consent; and discrimination/fairness. These domains guided but did not constrain analysis. Transcripts were iteratively coded, with new extracts prompting template revision. The final structure comprised four overarching themes: unprofessionalism, harassment, poor communication, and discrimination. Distinctions were maintained between reported events and participants' interpretations, and between breaches of conduct and moral dilemmas. Coding was iterative, disagreements discussed, and an audit trail maintained.

Trustworthiness was enhanced through several complementary strategies. Credibility was supported through member checking, whereby participants were invited during and after interviews to clarify meanings, elaborate experiences, and verify researchers' understanding of their narratives. Investigator triangulation was achieved through collaborative review and discussion of emerging codes and themes among members of the research team. Interpretations were also considered alongside relevant professional, regulatory, and bioethical literature to enhance analytical depth. Dependability was strengthened through maintenance of an audit trail documenting coding decisions, template revisions, and theme development throughout the analytic process.

Reflexivity was addressed continuously throughout the study. The research team comprised academics and clinicians working in dental education, medical education, and health professions education. Several members occupied leadership and supervisory positions within academic institutions and were familiar with the socio‐cultural and religious context of Pakistani healthcare education. While this insider positioning facilitated contextual understanding and interpretation of participants' narratives, it also created potential risks of normative assumptions regarding professionalism, ethics, and professional conduct. To minimize these influences, researchers engaged in ongoing reflexive discussions during study design, data collection, coding, analysis, and manuscript preparation. Team members critically examined how their educational, professional, cultural, and socio‐religious perspectives might shape interpretation of participants' reports. Regular peer debriefing and collaborative coding discussions were used to challenge assumptions, consider alternative explanations, and strengthen the credibility and confirmability of the findings.

Participants and researchers were not assumed to share a single worldview; socio‐cultural and religious context was treated as shaping moral reasoning. Analysis focused on how students perceived professional conduct in their own environment, interpreted alongside professional and bioethical frameworks, recognizing diversity within Islamic bioethics. These items are flagged in Appendix A for verification.

Ethical approval was obtained from the ASRB, Khyber Medical University (Dir/KMU‐EB/EP/000787). Participants received information sheets, gave written consent, and were assured confidentiality. Allegations are reported as participants' accounts, not independently verified. Copies of participant information and consent forms are in Appendix B.

Interviews were conducted in Urdu and English, depending on preference. All were audio‐recorded, transcribed verbatim, and translated into English. Urdu translations were verified, with culturally specific terms retained rather than replaced by generic biomedical terminology. The analytic team reviewed translations collectively to ensure socio‐cultural and religious nuances were preserved. This process safeguarded rigor by maintaining voluntariness, anonymity, and contextual meaning.

## 4 Results

Fifty students were approached and 37 participated (74.0%) using descriptive analysis [12]. Participants had a mean age of 20.9 ± 1.96 years (range 17–25). Twenty‐nine (78.4%) were female and eight (21.6%) males. Second‐, third‐ and fourth‐year students were represented, with fourth‐year students comprising the largest group (*n* = 14, 37.8%). Table 1 represents the demographic distribution below;

**Table 1:** Demographic distribution of the participants.

Variable	Category	*n* (%)/summary
Gender	Male Female	8 (21.6%) 29 (78.4%)
Age	Mean ± SD Range	20.9 ± 1.96 years 17–25 years
Year of study	Second year Third year Fourth year	11 (29.7%) 12 (32.4%) 14 (37.8%)

*Note:* The four final themes emerged from iterative Template Analysis. The initial four sensitizing domains were retained at a broad level, but their content was refined through coding. The final template therefore represents both deductive attention to professional responsibilities and inductive attention to what students actually described. Table 2 below presents the themes and subthemes identified.

**Table 2:** Themes and subthemes.

Theme	Subthemes
Unprofessionalism	1. Medical Negligence in Clinical Dental Practice 2. Harmful Clinical Interventions and Ethical Compromise
Harassment	1. Boundary Violations and Gender‐Based Harassment 2. Psychological Intimidation Within Academic Hierarchies
Poor communication	1. Deficiencies in Respectful Clinical Communication 2. Educational Communication and Supervisory Failures
Discrimination	1. Institutional Power Imbalances 2. Personal Targeting and Victimization

*Note:* The four themes were further refined into eight analytically derived subthemes through iterative Template Analysis. Rather than presenting isolated incidents, the subthemes represent broader patterns across participant narratives and demonstrate how different manifestations of professionalism challenges were experienced and interpreted within clinical learning environments.

## 5 Theme 1: Unprofessionalism

### 5.1 Subtheme 1.1: Medical Negligence in Clinical Dental Practice

Participants described incidents they perceived as compromising patient safety and professional responsibility. These accounts reflected concerns regarding infection control practices, failure to recognize or disclose clinical errors, and inadequate accountability when adverse outcomes occurred.

One participant described observing what they perceived as a serious breach of infection‐control standards:

I was present in the prosthodontic‐lab with my classmates, I saw one teacher that she took impression of one patient and use the same impression tray for another patient which is way so weird, I also talked to that teacher but she lied that it was not the one used for the other patient.

(Participant‐02)

The account illustrates how a seemingly routine clinical act was interpreted by a student as a patient‐safety and integrity concern. The concern is not only the technical act but also the perceived denial when the student raised it.

Similarly, participants reported concerns regarding the handling of clinical errors and professional accountability:

During a root canal, a student saw their teacher leave a canal unprepared, bypass X‐rays because of ‘not enough time,’ and perforate a tooth. Without taking an X‐ray, the teacher only administered medication when the patient returned in pain. Later, the patient visited another clinic where the student was interning; a successful re‐RCT was performed, the floor was fixed, and an X‐ray identified the problem. ‘I won't be going to him or ever referring any patient to him,’ the student said in closing.

(Participant‐37)

This narrative links technical error to accountability, disclosure, and trust. The student's final statement also demonstrates how observed faculty behavior may influence students' future willingness to trust or recommend clinicians.

### 5.2 Subtheme 1.2: Harmful Clinical Interventions and Ethical Compromise

Participants also reported situations in which educational or institutional pressures appeared to take precedence over patient welfare. These narratives highlighted perceived failures of empathy and concerns regarding treatment decisions that students considered ethically questionable.

One participant reflected on the distress of watching a patient undergo repeated unsuccessful attempts at local anesthesia administration:

I understand that we as students need to learn anesthesia on our own, but to watch a patient being pricked multiple times because the student is nervous and the teacher wants her to learn perfectly while the patient sits there helplessly is heartbreaking. I felt bad seeing. But would like it if someone came up with one. Because we all vow ‘do no harm’.

(Participant‐10)

The participant connected clinical teaching with non‐maleficence and compassion. The ethical tension lies between providing learning opportunities and preventing avoidable patient discomfort, highlighting the importance of supervision and timely intervention.

Participants also reported circumstances in which they felt pressured to engage in treatment they considered ethically inappropriate:

I was told by an instructor during end of rotation to restore arrested caries after being assigned a patient with irreversible pulpitis. A shallow cavity made the restoration difficult, who felt under pressure to conform. The teacher helped me by framing it as ‘getting rid of the patient.’ Because they compromised healthy tooth structure, I felt guilt and wonders if they done more harm than good and who was at responsibility.

(Participant‐03)

This extract demonstrates how students may experience moral discomfort when hierarchical authority appears to conflict with patient welfare. The ethical concern therefore extended beyond the clinical decision itself to include the circumstances under which the decision was made.

## 6 Theme 2: Harassment

### 6.1 Subtheme 2.1: Boundary Violations and Gender‐Based Harassment

Participants described experiences in which professional boundaries were allegedly violated through inappropriate comments, physical contact, and misuse of authority. Such behaviors were frequently linked to hierarchical relationships that discouraged reporting.

One participant reported inappropriate physical contact by a faculty member:

One of my male Professor used to harass his female students by touching inappropriately during clinical work.

(Participant‐21)

This account describes an alleged boundary violation occurring within a teacher‐student relationship. Given the study design, the narrative is presented as a participant's account rather than a verified event.

Similarly, another participant described unwanted comments and behavior from a senior colleague:

I was at the operative clinic in my institute where a senior (male) of ours was examining my class mate (female). Time to time, he'd touch her cheeks and pass comments like you're getting chubbier or like how chubby cheeks you have and he also held her necklace. My fellow felt uncomfortable and asked for a female senior fellow for guidance but he didn't let her ask another senior. He's a well‐known senior and we're afraid to complain about him to the authorities.

(Participant‐31)

The account combines inappropriate physical contact, comments about appearance, and fear of reporting. The perceived power imbalance appears central to the ethical significance of the experience.

### 6.2 Subtheme 2.2: Psychological Intimidation Within Academic Hierarchies

Beyond direct interactions, several participants described a workplace culture characterized by intimidation, fear, and psychological pressure.

One of my colleagues was emotionally harassed by the senior faculty time and again, the ‘aged doctor’ took advantage of this imbalance and fostered an atmosphere that was conducive to misconduct. This environment is intimidating in and of itself.

(Participant‐36)

The participant frames repeated intimidation as a systemic rather than purely interpersonal issue, suggesting that professionalism is shaped by institutional culture as well as individual behavior.

## 7 Theme 3: Poor Communication

### 7.1 Subtheme 3.1: Deficiencies in Respectful Clinical Communication

Participants frequently linked professionalism to respectful communication and patient‐centered care. Several accounts described communication practices perceived as disrespectful, coercive, or inconsistent with informed decision‐making.

One participant described a faculty member shouting at a patient:

I was in clinical areas and my staff teacher was there examining a patient. Suddenly she started shouting at her, whatever the reason was. That was a horrific thing to encounter. It looked like she need to take history classes again.

(Participant‐19)

The student interpreted shouting at a patient as incompatible with respectful communication and as a negative model of professional conduct.

Participants also expressed concern about witnessing potentially forceful treatment of vulnerable patients:

During my rotation in OMFS department, I saw a postgraduate trainee in the OMFS department, pulling out a child's teeth with force, the child yelled in agony. The trainee continued the process without showing empathy or pausing, seeming agitated and hostile. I wanted to step in since they thought the scenario was extremely immoral, but I felt helpless because of the seniority structure.

(Participant‐22)

This extract demonstrates how hierarchical relationships may inhibit students from speaking up even when they perceive patient welfare to be compromised.

Participants additionally described concerns regarding informed consent practices:

A patient arrived for a simple tooth extraction during my rotation in OMFS‐department. A house‐officer continued extraction without outlining potential dangers, such as dry socket. The house‐officer never inquired about an underlying medical condition that elevated the patient's risk after the treatment. Having never been given the opportunity to make an educated decision, the patient felt deceived.

(Participant‐30)

The participant interpreted the absence of risk disclosure and relevant history‐taking as a failure of informed consent and respect for patient autonomy.

### 7.2 Subtheme 3.2: Educational Communication and Supervisory Failures

Several students described communication challenges within the educational environment itself, including insufficient supervision, unclear instructions, and ineffective teaching practices.

One participant reported receiving criticism despite not being given adequate guidance:

I was in Medicine ward and having an end of rotation test. Everyone else was busy doing their work. My patient whom I need to examine was a last stage cancer patient and her attendants were sitting near her. My supervisor asked me to take history and do examination but did not give any instruction. At the end he scolded me Infront of the patient that I did not follow the SOP's.

(Participant‐16)

This account demonstrates how inadequate supervision can undermine both student learning and professional dignity.

Another participant described frustrations with online teaching practices:

In online classes they just present their PowerPoint presentation, without proper explanations. They give it in 1 short‐lecture, without any question‐answer session. Where to ask questions from them no proper teaching. I just hate these online classes. In pharma no proper drug interactions are taught in online classes.

(Participant‐06)

For this participant, ineffective teaching practices were perceived as jeopardizing the development of professional competence and readiness for clinical practice.

## 8 Theme 4: Discrimination

### 8.1 Subtheme 4.1: Institutional Power Imbalances

Participants reported experiences suggesting unequal treatment within institutional settings, particularly where authority structures or professional status appeared to influence fairness and respect.

One participant described feeling devalued because of their professional identity as a dental student:

I felt dental students compared to MBBS and degraded. By the derogatory remarks of the doctors emphasizing you are not doctors you are merely dentist. No issue can be solved until we get over this mental torture that we face from day one although we have chosen this field over MBBS then why all this hatred. It's a humble request if this platform really works and hear our voices thanks a lot!

(Participant‐04)

The account highlights how status hierarchies between healthcare professions may undermine collegiality, respect, and professional identity formation.

Another participant described concerns regarding alleged withholding of house‐officer stipends:

This issue is besides patients, but it is an illegal act that needs to be addressed. It's about the treatment of house‐officers. Government rules allows a set amount of stipend. That amount is not given to the house‐officers of XYZ College. We do not know where they keep the rest of the money, but every time, around 10,000rs are withheld. Yet no complaint could be made. Regulatory bodies should take action against this unprofessional & ethical act.

(Participant‐10)

The participant interpreted this practice as a misuse of institutional authority and an issue requiring accountability. As with other findings, this allegation was not independently verified.

### 8.2 Subtheme 4.2: Personal Targeting and Victimization

Students also described experiences in which personal circumstances or previous interactions appeared to lead to repeated negative treatment.

One participant reported feeling singled out because of a family association:

During my viva. I was reminded of my father's famous position in the dental college with a few derogatory remarks were made on a wrong answer, go ask your father the correct answer he will tell you. Another professor while taking viva reminding of how my father was as their supervisor and tried to scare me during my viva.

(Participant‐11)

The participant perceived personal relationships as being used in a manner that undermined fairness and psychological safety during assessment.

Similarly, another participant described experiences of repeated targeting after missing a university activity:

We have a teacher in our university who is always angry, when I was in 2nd year, she was my mentor she asked me to participate in a community outreach program but due to some illness I couldn't attend, from that day whenever she used to see me, she targets me by passing derogatory remarks Infront of other faculty‐members and my class‐mates.

(Participant‐17)

This account suggests that students may perceive isolated disagreements as developing into longer‐term patterns of adverse treatment, potentially affecting confidence, belonging, and willingness to seek support.

## 9 Discussion

The findings show that students' concerns were distributed mainly across four domains unprofessionalism, harassment, poor communication and discrimination, but these domains overlap conceptually. Infection‐control failures, lack of empathy, inadequate consent and poor supervision all implicate non‐maleficence and beneficence; harassment and discrimination implicate dignity and justice; and failure to disclose or acknowledge error implicates honesty, accountability and trust. The central finding is therefore not simply that isolated ‘bad behaviors’ occur, but that students encounter a gap between formal professional ideals and the enacted norms of the clinical learning environment.

A useful framework for interpreting this gap is provided in a study emphasizes professional morality requires more than memorizing principles [13]. General moral norms need specification in concrete contexts, and conflicting considerations may need balancing. A student who watches a novice struggle with local anesthesia, for example, may recognize the educational value of supervised practice while simultaneously recognizing the patient's claim not to be subjected to avoidable repeated harm. The ethical response is not to abandon training or non‐maleficence, but to specify the conditions under which trainee learning can proceed safely appropriate supervision, proportionality, patient consent and readiness to intervene.

The same reasoning applies to informed consent. The participant account concerning extraction illustrates a direct failure to disclose relevant risks and obtain an adequately informed decision. In Pakistan, family participation and relational decision‐making may be important, but contextual sensitivity should not be equated with removal of the patient's voice. Research in Pakistan has described tensions between individual autonomy and family involvement, suggesting that culturally responsive consent should accommodate legitimate family participation while preserving the competent patient's rights [14]. Thus, contextualization should refine the practice of autonomy rather than erase it.

The findings also complicate a simplistic contrast between ‘Western’ professionalism and ‘Islamic’ values. Islamic bioethical scholarship identifies substantial compatibility between contemporary principles such as beneficence, non‐maleficence and justice and higher objectives concerned with preservation of life and welfare [15]. At the same time, Islamic ethical reasoning is internally diverse. In the present study, it is therefore more defensible to speak of a Pakistani socio‐cultural and religious context that may shape how universal professional duties are interpreted and enacted, rather than claiming that a single Islamic position determines professional conduct.

This specification perspective is particularly important for truth‐telling, disclosure and social harmony. The goal should not be to portray Pakistani social harmony as a justification for deception or concealment. Instead, professional truthfulness can be understood as a duty that must be communicated with sensitivity to family relationships, language, power and the patient's preferences. The ethical task is to protect dignity, informed decision‐making and trust while allowing context to shape the process of communication. This approach avoids both uncritical universalism and cultural relativism.

The choice to focus on dental professionalism is also justified by the distinctive structure of dental education according to previous study [16]. Dental students frequently learn through direct patient contact, procedural performance, close supervision and visible role modeling. The FDI's dental‐specific ethical principles and PM & DC's regulatory responsibilities provide a profession‐specific normative reference point against which students can interpret clinical conduct [3] [7]. This does not mean that the problems identified are unique to dentistry. Harassment, discrimination, poor communication and abuse of hierarchy are likely to occur across health professions. Rather, the dental focus defines the scope of inference and provides a coherent starting point for future interprofessional research.

Although participants were recruited from both public and private dental institutions, the study was not designed as a comparative analysis of institutional sectors. Professionalism concerns were reported across both settings, and the available data did not allow robust conclusions regarding sector‐specific differences. Future research employing comparative sampling strategies may explore whether institutional culture, resources, and organizational structures shape students' experiences of professional conduct differently in public and private settings.

The findings of this study, have implications for the hidden curriculum [17]. Students do not learn professionalism only from lectures; they learn what is tolerated, rewarded and normalized in clinical settings. When a faculty member is perceived to disregard consent, empathy or accountability, students may receive a contradictory message about what professional conduct actually means. Conversely, respectful communication, transparent error management and safe opportunities to speak up can make formal ethics education credible according to number of studies [18] [19].

The results of the current study, therefore support a longitudinal model of professionalism education in which ethical concepts are introduced early, revisited during clinical years, explicitly linked to observed cases, and assessed through multiple methods. Another study suggests; faculty development is equally important as students cannot be expected to internalize standards that are repeatedly contradicted by role models [20][21]. Institutions should also establish confidential and non‐retaliatory reporting pathways for harassment and serious professional concerns.

## 10 Conclusion

Undergraduate dental students in this study described experiences and observations that they perceived as breaches of professional conduct across four interconnected domains: unprofessionalism, harassment, poor communication and discrimination. The findings suggest that professionalism is shaped not only by formal curriculum but also by the clinical environment, power relationships and role modeling. Strengthening professionalism in Pakistani dental education therefore requires longitudinal ethics education, explicit discussion of context‐sensitive moral reasoning, faculty role‐modeling, effective supervision, protection of patient rights, and safe mechanisms through which students can raise concerns.

A context‐sensitive approach should not weaken universal commitments to dignity, non‐maleficence, beneficence, justice and informed decision‐making. Instead, it should use specification and reflective ethical reasoning to determine how these commitments can be enacted appropriately within Pakistan's social, legal and religious context.

## 11 Limitations

The study used a qualitative design with purposive sampling; findings provide contextual insight rather than statistical generalization. Thirty‐seven participants from two dental colleges in Peshawar limit transferability to other regions, institutions, and professions. Recruitment via email, WhatsApp, Facebook, and notice boards may have introduced self‐selection, while fear of identification could have deterred others; non‐response bias cannot be assessed. Focusing on dental professionalism provides coherence but restricts claims about other health professions, highlighting the need for interprofessional studies. Institutional culture may also have shaped disclosures, suggesting value in multi‐site research. The team's insider positioning aided interpretation but risked hierarchy‐related bias, addressed through reflexive procedures, though some COREQ reflexivity items (e.g., interviewer identity) require verification. Finally, details on interview duration, setting, language, transcription, and translation were missing in the original manuscript and should be completed from field records to ensure reproducibility and cultural accuracy. Although, the participants were recruited from both public and private institutions, this study was not designed to compare these settings systematically. Therefore, conclusions regarding differences between institutional sectors cannot be drawn from the present data.

## 12 Clinical Relevance

The study suggests that ethical competence in dentistry depends on the interaction between formal teaching and the clinical learning environment. Educators should recognize their role as professional role models, strengthen supervision and consent practices, create psychologically safe reporting pathways, and explicitly discuss how universal professional duties are applied within Pakistan's socio‐cultural context.

## Author Contributions

**Sarah Ali:** conceptualization, methodology, formal analysis, writing – original draft, writing – review and editing. **Syed Ali Ayub:** software, investigation, supervision, writing – review and editing. **Shahzad Ahmad:** funding acquisition, resources. **Riaz Shahbaz Janjua:** data curation, visualization, project administration, resources. **Jamilah Janjua:** validation. **Shazia Iqbal:** validation, formal analysis.

## Ethics Statement

Ethical approval was granted by the Advanced Studies Review Board (ASRB), Khyber Medical University Research and Ethics Board (Dir/KMU‐EB/EP/000787). Written informed consent was obtained from all participants.

## Conflicts of Interest

The authors declare no conflicts of interest.

## Transparency Statement

I Dr. Sarah Ali, corresponding author, on behalf of all the authors affirms that this manuscript is an honest, accurate, and transparent account of the study being reported; that no important aspects of the study have been omitted; and that any discrepancies from the study as planned (and, if relevant, registered) have been explained. The manuscript is original, has not been published previously, and is not under consideration elsewhere.

## Data Availability

The data that support the findings of this study are available from the corresponding author upon reasonable request, subject to applicable ethical and confidentiality restrictions.

## 1 COREQ. 32‐Item Reporting Checklist

The checklist is included to demonstrate transparent reporting of qualitative interviews. It is based on Tong, Sainsbury and Craig (2007). Items marked “Author verification required” were not explicitly documented in the source manuscript and should be completed from the original study records before submission.

**Table:**

S. no	Domain/item	COREQ requirement	Status in revised manuscript
1	Interviewer/facilitator	Identity of interviewer	Author verification required
2	Credentials	Academic/professional credentials	Partly reported; interviewer‐specific details require verification
3	Occupation	Occupation at study time	Partly reported through author affiliations; interviewer requires verification
4	Gender	Gender of interviewer	Author verification required
5	Experience/training	Training/experience in qualitative interviewing	Author verification required
6	Relationship established	Relationship with participants before study	Author verification required
7	Participant knowledge	What participants knew about interviewer	Partly reported; verify
8	Interviewer characteristics	Potential characteristics/influences	Addressed in reflexivity; individual interviewer requires verification
9	Methodological orientation	Methodological/theoretical orientation	Qualitative narrative inquiry; Template Analysis
10	Sampling	How participants were selected	Purposive sampling reported
11	Method of approach	How participants were approached	Email, WhatsApp, Facebook and notice boards
12	Sample size	Number approached and participating	50 approached; 37 participated
13	Non‐participation	Reasons for non‐participation	Not systematically documented; stated as limitation
14	Setting	Where data collected	Exact setting requires verification
15	Presence of non‐participants	Others present during interviews	Author verification required
16	Description of sample	Participant characteristics	Age, gender and year reported
17	Interview guide	Interview guide development	Five questions; expert review and prompts reported
18	Repeat interviews	Repeat interviews	Author verification required
19	Audio/visual recording	Recording method	Author verification required
20	Field notes	Field notes	Author verification required
21	Duration	Interview duration	Author verification required
22	Data saturation	Saturation/discussion of stopping	Author verification required
23	Transcripts returned	Participant transcript checking	Clarification/member checking reported; transcript return requires verification
24	Number of data coders	Who coded data	Team discussion reported; individual coding allocation requires verification
25	Coding tree	Description of coding tree	A priori domains and final themes reported
26	Derivation of themes	Deductive/inductive process	Both specified; Template Analysis
27	Software	Software used	Author verification required
28	Participant checking	Participant feedback on findings	Clarification/member checking reported; verify exact procedure
29	Quotations	Supporting quotations	Included throughout results
30	Data consistency	Consistency between data and findings	Analytic descriptions added
31	Clarity of major themes	Major themes clearly presented	Four themes reported
32	Clarity of minor themes	Subthemes clearly presented	Subthemes reported in Table 2

## 1 Participant Information and Informed Consent Form (Reporting Copy)

Important: This appendix is provided to show the information that should accompany the manuscript. The authors should confirm that it reproduces the ethics‐approved form actually used in 2020–2021 before submission.

**Study title:** Navigating Professional Code of Conduct Among Undergraduate Dental Students in Pakistan: A Qualitative Study.

**Purpose:** You are invited to take part in a research study exploring dental students' experiences and observations of professional conduct during clinical/workplace learning.

**Voluntary participation:** Participation is voluntary. You may decline to participate or stop the interview without penalty.

**Confidentiality:** Your name and other direct identifiers will not be used in publications. Quotations will be identified by a participant number.

**Potential sensitivity:** Some questions may involve uncomfortable experiences, including harassment, discrimination or concerns about professional conduct. You may decline to answer any question.

**Data use:** Information collected will be used for research and may be reported in anonymized form in academic publications.

**Consent statement:** I have read and understood the information provided. I have had the opportunity to ask questions. I understand that participation is voluntary and that I may withdraw according to the study procedures. I agree to participate in the interview.

Participant name: _________

Signature: _______

Date: ___________

Researcher name: _________

Signature:________________
